# Metabolism of 3-[5'-deoxy-5'-(dimethylarsinoyl)-β-ribofuranosyloxy]-2-hydroxypropylene glycol in an artificial digestive system

**DOI:** 10.1016/j.heliyon.2019.e02079

**Published:** 2019-07-19

**Authors:** Akihisa Hata, Momoko Hasegawa, Takenori Yamauchi, Yuki Otomo, Motofumi Miura, Kenzo Yamanaka, Yuko Yamano, Noboru Fujitani, Ginji Endo

**Affiliations:** a Faculty of Veterinary Medicine, Okayama University of Science, 1-3 Ikoino-oka, Imabari, Ehime, 794-8555, Japan; b Department of Medical Risk Management, Graduate School of Risk and Crisis Management, Chiba Institute of Science, 15-8 Shiomi-cho, Choshi, Chiba, 288-0025, Japan; c Department of Hygiene and Preventive Medicine, School of Medicine, Showa University, 1-5-8 Hatanodai, Shinagawa-ku, Tokyo, 142-8555, Japan; d Laboratory of Molecular Chemistry, School of Pharmacy, Nihon University, 7-7-1 Narashinodai, Funabashi, Chiba, 274-8555, Japan; e Laboratory of Environmental Toxicology and Carcinogenesis, School of Pharmacy, Nihon University, 7-7-1 Narashinodai, Funabashi, Chiba, 274-8555, Japan; f Biomedical Science Examination and Research Center, Okayama University of Science, 1-3 Ikoino-oka, Imabari, Ehime, 794-8555, Japan; g Osaka Occupational Health Service Center, Japan Industrial Safety and Health Association, 2-3-8 Tosabori, Nishi-ku, Osaka, 550-0001, Japan

**Keywords:** Food Analysis, Environmental Health, Environmental Toxicology, Toxicology, Public Health, Food Science, Arsenic, Arsenosugar, Seafood, Intestinal bacteria, Thiolation

## Abstract

Seaweeds contain large amounts of organoarsenic compounds, mostly arsenosugars (AsSug) and arsenolipids (AsLipid). AsSug is mainly metabolized into dimethylarsinic acid (DMA^**V**^) in humans. However, this metabolic process is not well understood. We investigated the metabolism of an AsSug, 3-[5'-deoxy-5'-(dimethylarsinoyl)-β-ribofuranosyloxy]-2-hydroxypropylene glycol (AsSug328), in the gastrointestinal tract using an *in vitro* artificial gastrointestinal digestion system. AsSug328 was incubated with gastric juice for 4 h, with bile-pancreatic juice for 0.5 h, and finally with enteric bacteria solution for 24 h. The conversion of arsenic compounds after artificial digestion was analyzed by HPLC-ICP-MS and HPLC-ESI-Q-TOF-MS. Our results show that artificial gastrointestinal digestion converted AsSug328 into thio-AsSug328. However, no formation of DMA^**V**^ was detected. Under the artificial digestion system, the 5-deoxyribofuranose structure of AsSug was maintained. Therefore, AsSug should be absorbed in the intestinal tract after its sugar moiety is partially decomposed. They are then possibly metabolized to DMA^**V**^ in the liver and subsequently excreted through urine.

## 1 Introduction

Seafood is known to be abundant in arsenic compounds [1, 2, 3]. Seaweeds contain arsenic compounds, most of which are arsenosugars (AsSugs) and arsenolipids (AsLipids) [4, 5, 6, 7, 8, 9]. The International Agency for Research on Cancer (IARC) has placed arsenic and inorganic arsenic in Group 1 (carcinogenic to humans), and monomethylarsonic acid (MMA^V^) and dimethylarsinic acid (DMA^V^) in Group 2B (these are possibly carcinogenic to humans) [10]. However, IARC has not assessed the carcinogenicity of AsSug and AsLipid because of the lack of information about their metabolism and intermediates formed.

In previous studies, AsSugs were reported to be metabolized into dimethylated arsenic compounds, such as DMA^V^, thio-dimethylarsenoethanol (thio-DMAE^V^), and thio-dimethylarsenoacetic acid (thio-DMAA^V^), for excretion into the human urine [11, 12, 13]. A noteworthy fact is that dimethylmonothioarsinic acid (DMMTA^V^) is a trace arsenical that is excreted in human urine after ingestion of AsSug [12]. DMMTA^V^ is as genotoxic and cytotoxic as dimethylarsinous acid (DMA^III^), which is a known carcinogen [14]. Intestinal bacteria, like *Escherichia coli*, that metabolize DMA^V^ to DMMTA^V^, are hypothesized to play an important role in the carcinogenicity of DMA^V^ [15]. Therefore, it is necessary to clarify the processes for the degradation of AsSug into DMA^V^ in the digestive system.

A previous *in vivo* metabolic study using 3-[5'-deoxy-5'- (dimethylarsinoyl)-β-ribofuranosyloxy]-2-hydroxypropylene glycol (AsSug328) showed DMA^V^ is excreted into the human urine after the ingestion of AsSug328 [11, 12, 13]. However, the mechanism of degradation of AsSug328 to DMA^V^ during digestion process is not clear. In the present study, we investigated the degradation of AsSug328, *in vitro*, using artificial digestive juices consisting of gastric juice, bile-pancreatic juice, and intestinal bacterial flora obtained from a healthy adult human. AsSug328 is one of the most typical AsSug, detected universally in seaweeds. Moreover, AsSug328 has a simpler side chain structure than other AsSugs detected in seaweeds [4, 8]. Thus, we chose AsSug328 as a model for the intake of AsSugs to clarify its metabolism in the digestive system.

This study demonstrates that *in vitro* digestion of AsSug328, using *in vitro* artificial digestion system, could provide vital information about the actual digestion of such compounds in the human gut. This would be helpful in assessing the toxicity of organoarsenic compounds present in seafood.

## 2 Materials and methods

### 2.1 Ethical considerations

The study plan was approved by the Ethical Committee for Medical and Health Research Involving Human Subjects of Chiba Institute of Science (approval number 22-4). Informed consent was taken from the subject for using urine and fecal samples.

### 2.2 Reagents

Sodium arsenite (As^III^), sodium arsenate (As^V^), MMA^**V**^, and arsenobetaine were purchased from Wako Pure Chemical Industries (Osaka, Japan). DMA^**V**^ was obtained from Tri Chemical Laboratory (Yamanashi, Japan). AsSug328 was synthesized following the procedure described by Traar and Francesconi [16]. The concentration of each arsenic compound was considered as the elemental arsenic concentration. The enzymes, bile, and pancreatic salts used in the *in vitro* digestion were purchased from Sigma Chemical Co. (St. Louis, MO, USA); these were pepsin (porcine, P-7000), pancreatin (porcine, P-1750), and bile extract (porcine, B-8631). The growth cofactor for anaerobic bacteria, hemin (porcine, A-11165), was purchased from Alfa Aesar Co. (Lancashire, UK).

For the mobile phase used in high performance liquid chromatography (HPLC), we used nitric acid (HNO_3_, Ultrapure; Kanto Chemical, Tokyo, Japan), ammonium nitrate (NH_4_NO_3_, JIS Special Grade; Wako Pure Chemical), acetic acid [CH_3_COOH, for liquid chromatography–mass spectrometry (LC/MS) analysis; Wako Pure Chemical Industries], ammonium acetate [CH_3_CO_2_NH_4_, for HPLC analysis (99%); Sigma-Aldrich Japan, Tokyo, Japan], ammonium hydrogen carbonate (NH_4_HCO_3_, for LC/MS analysis; Sigma-Aldrich Japan), and methanol (CH_3_OH, for LC/MS analysis; Wako Pure Chemical Industries). Ultrapure water was prepared using a MilliQ-ICP/MS Ultrapure Water Purification System (Millipore, Tokyo, Japan).

### 2.3 Artificial digestive juice

The artificial gastric juice was constituted with reference to the Japanese Pharmacopoeia and as described previously by Marques *et al.* [17, 18]. The artificial gastric juice (pH 1.2) consisted of 200 mg of NaCl, 10 mg of pepsin, and 0.7 mL of 37% HCl in 100 mL of Milli-Q water. The composition of artificial bile-pancreatic juice was in accordance with that described by Laparra *et al.* [19]. The bile-pancreatic juice (pH 8.2) contained 2.5 g of bile extract and 400 mg of pancreatin in 100 mL of 0.1 M NaHCO_3_.

The solution of enteric bacteria was prepared from fresh feces obtained from a healthy male adult (35 years old) after restriction of seafood intake for five days; this individual had no history of antibiotic treatment in the year preceding this study. We confirmed beforehand that he could metabolize AsSug to DMA^V^ by feeding seaweed. Under the seafood-restricted diet, 47% of arsenic content in urine was in the form of DMA^V^ after seaweed ingestion, which included AsSug328 and AsSug482 but not DMA^V^, MMA^V^, and inorganic arsenic. Fresh feces (7 g) were suspended in sterile phosphate buffer (pH 7.2) and saburra was removed by filtration through a gauze. The filtrate was centrifuged at 3,000 *× g* for 10 min at room temperature; the sediment that was obtained was suspended in phosphate buffer (pH 7.2) and the volume was made up to 20 mL. This process took 20 min and was conducted just before use. The suspension, thus obtained, was used as the enteric bacteria solution. Next-generation sequencing analysis was used to detect the presence of the members of the Bacteroidetes (73.4%) and Firmicutes (24.3%) phyla in the enteric bacteria solution; these bacteria are obligate anaerobes present as enterobacterial flora in healthy humans.

### 2.4 In vitro gastrointestinal digestion of AsSug328

The incubation time for each phase of reaction in the artificial digestive system was set with reference to the human physiological digestion time [20]. The procedure for the shift from gastric phase to intestinal phase was according to the method described by Laparra *et al.* [19]. Five milliliters of 8 mg As/L AsSug328 solution (sample A) was dispensed into screw cap vials, to which 0.5 mL of the freshly prepared artificial gastric juice was added and the mixture was incubated in a constant temperature incubator shaker (100 rpm, Bio-Shaker BR-40LF, TAITEC, Saitama, Japan) at 37 °C for 4 h (sample B). After the pH of the gastric digests was raised to 5 by the addition of 1 M NaHCO_3_, 0.7 mL of artificial bile-pancreatic juice was added and the pH was adjusted to 7.2 by the addition of 0.5 M NaOH. The volume was made up to 7 mL with Milli-Q water and the solution was shaken at 37 °C for 0.5 h (sample C). Freshly prepared enteric bacteria solution (2 mL) and hemin (5 μg/mL in the final sample volume) were added to the intestinal digests after deoxidation by bubbling of argon. The volume was made up to 10 mL with phosphate buffer (pH 7.2) and shaken at 37 °C for 24 h (sample D). To oxidize the sulfurated arsenic compound in sample D, 0.9 mL of the enteric bacteria-treated sample was added to 0.1 mL of 30% (w/v) H_2_O_2_ (sample E). The sampling was done at the end of each digestive phase. The collected samples were filtered through a 0.2-μm PTFE membrane filter (Millex-LG, Millipore Corp., MA, USA) and analyzed immediately.

### 2.5 Total As analysis

Total As concentrations were determined by iCAP Qc ICP-MS (Thermo Fisher Scientific, MA, USA). The ICP-MS assay was conducted under the following instrumental conditions: RF power, 1550 W; plasma argon gas flow rate, 14 L/min, auxiliary argon gas flow rate, 0.8 L/min, and nebulizer argon gas flow rate, 1.0 L/min. The sample, diluted 500-fold with 0.5% nitric acid, was used for ICP-MS analysis.

### 2.6 HPLC-ICP-MS analysis

We used two HPLC conditions because it is difficult to completely separate several arsenic species in a sample using a single condition. We used the anion and cation exchange methods for speciation analysis of arsenic in biological sample.

A Dionex ICS-5000 system (Thermo Fisher Scientific) was used to separate the different arsenic species, and iCAP Qc ICP-MS was used to detect the presence of arsenic compounds. A Hamilton PRP-X100 anion exchange column (150 × 4.1 mm inner diameter; Hamilton, Reno, NV, USA) was used under the following analytical conditions: mobile phase, 20 mM NH_4_HCO_3_ (at pH 9.0; adjusted with NH_3_ solution):CH_3_OH (97:3); flow rate, 1.2 mL/min; column temperature, 40 °C; and injection volume, 50 μL. A Shodex RSpak NN-614 cation-exchange column (150 × 6.0 mm inner diameter; Showa Denko, Tokyo, Japan) was used under the following analytical conditions: mobile phase, 20 mM CH_3_COOH-CH_3_CO_2_NH_4_ (at pH 4.2):CH_3_OH (90:10); flow rate, 0.7 mL/min; column temperature, 40 °C; injection volume, 50 μL. The sample, diluted 50-fold with MilliQ-H_2_O, was used for HPLC-ICP-MS analysis.

A defect of this anion exchange method is that it cannot separate AsSug328 and AsSug254. On the other hand, the defect of the cation exchange method is that it cannot separate thio-AsSug328 and DMA^V^. The purpose of this study was to investigate the process of breakdown of AsSug328 into DMA^V^. An analytic condition that cannot separate DMA^V^ from other arsenics might lead to serious errors. Therefore, in this study, anion exchange was used as the main analytical method, and cation exchange was used as an auxiliary method.

### 2.7 HPLC-ESI-Q-TOF-MS analysis

For identification of arsenic species in the AsSug328 eluent, we ascertained the accurate ion mass number and MS/MS fragment ion pattern using ESI-Q-TOF-MS. The arsenic species that we targeted are listed in Table 1. The chemical structures of the arsenic species described in Table 1 were derived from previous studies [12, 21], and the accurate mass number was calculated based on these chemical formulae.

**Table 1:** Molecular formulae of arsenic compounds targeted in the HPLC-ESI-Q-TOF-MS analysis.

Name	Abbreviation	Rational formula
Oxo-arsenosugar-254	AsSug 254	C_7_H_15_AsO_5_
Oxo-arsenosugar-328	AsSug 328	C_10_H_21_AsO_7_
Oxo-arsenosugar-391	AsSug 391	C_10_H_22_AsO_8_SN
Oxo-arsenosugar-392	AsSug 392	C_10_H_21_AsO_9_S
Oxo-arsenosugar-408	AsSug 408	C_10_H_21_AsO_10_S
Oxo-arsenosugar-482	AsSug 482	C_13_H_28_AsO_12_P
Thio-arsenosugar-328	thio-AsSug328	C_10_H_21_AsO_6_S
Monomethylarsonic acid	MMA^V^	CH_5_AsO_3_
Dimethylarsinic acid	DMA^V^	C_2_H_7_AsO_2_
Thio-dimethylarsinic acid	thio-DMA^V^	C_2_H_7_AsOS
Oxo-dimethylarsenoacetic acid	oxo-DMAA^V^	C_4_H_9_AsO_3_
Thio-dimethylarsenoacetic acid	thio-DMAA^V^	C_4_H_9_AsO_2_S
Oxo-dimethylarsenoethanol	oxo-DMAE^V^	C_4_H_11_AsO_2_
Thio-dimethylarsenoethanol	thio-DMAE^V^	C_4_H_11_AsOS
Trimethylarsine oxide	TMAO^V^	C_3_H_9_AsO
Trimethylarsine sulfide	TMAS^V^	C_3_H_9_AsS
Oxo-analog of unknown arsenic metabolite	oxo-U	C_6_H_15_AsO_3_
Thio-analog of unknown arsenic metabolite	thio-U	C_6_H_15_AsO_2_S

We used Xevo G2-XS (Waters, MA, USA) system for ESI-Q-TOF-MS. The following parameters were set for the ESI-Q-TOF-MS analysis: capillary voltage, 2.9 kV (positive mode); ion source temperature, 120 °C; desolvation nitrogen gas temperature, 450 °C; desolvation gas flow, 13.3 L/min. The Xevo G2-XS was connected to an Acquity Ultra Performance Liquid Chromatography (UPLC) system (Waters). We injected 10 μL of each sample into the Acquity UPLC system using cation and anion exchange columns. An NN-614 cation-exchange column (150 × 6.0 mm inner diameter) was used under the following conditions: 20 mM CH_3_COOH-CH_3_CO_2_NH_4_ (at pH 4.2): CH_3_OH (90:10); flow rate, 0.7 mL/min; column temperature, 40 °C. A Hamilton PRP-X100 anion exchange column (250 × 2.1 mm inner diameter) was used under the following conditions: 20 mM NH_4_HCO_3_ (at pH 9.0; adjusted with NH_3_ solution):CH_3_OH (97:3); flow rate, 0.3 mL/min; column temperature, 40 °C. The undiluted sample was used for HPLC-ESI-Q-TOF-MS analysis.

## 3 Results

### 3.1 Confirmation of the composition of AsSug328

**Fig. 1:**
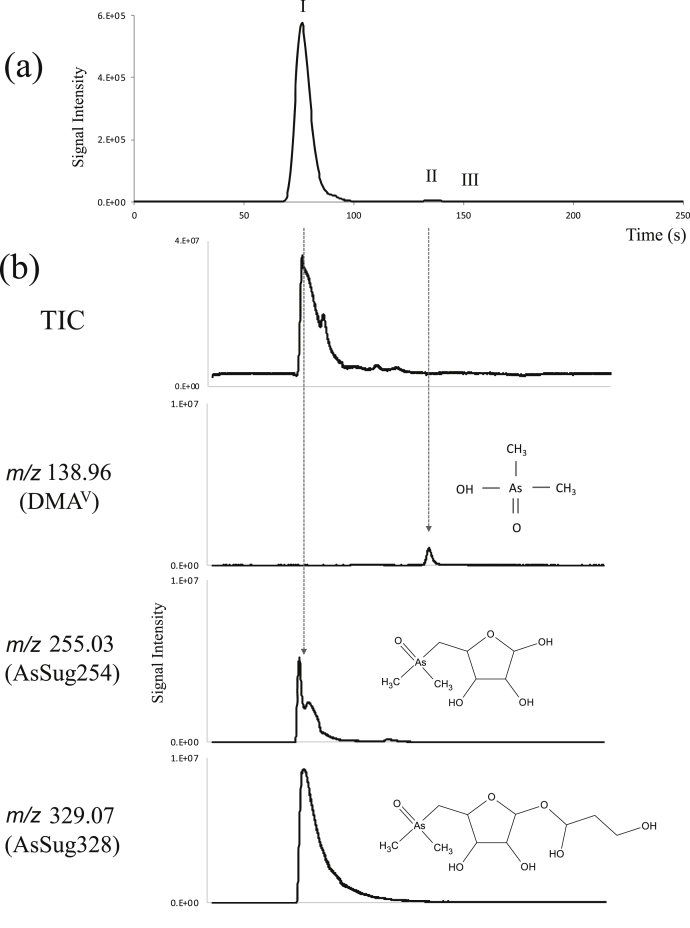
(a) HPLC-ICP-MS and (b) HPLC- ESI-Q-TOF-MS chromatograms of AsSug328 used in this study (sample A). The arsenic compounds were separated by anion-exchange column chromatography on a PRP-X100 column.

The AsSug328 solution used in this study (sample A) showed three arsenic peaks (I–III) in HPLC-ICP-MS analysis using the anion-exchange column PRP-X100 (Fig. 1). The recovery rate of As [(sum of speciation As/Total As) × 100] was 98%. The retention times of peaks I and II matched those of AsSug328 and DMA^V^, respectively. The retention time of peak III did not match the retention time of any of the standards used. The accurate ion mass number and MS/MS fragment ion pattern, as determined by the HPLC- ESI-Q-TOF-MS analysis, revealed that peak I was of AsSug328 and AsSug254, and peak II was of DMA^V^ (Fig. 1). Peak III could not be identified. The arsenic compounds described in Table 1, excluding AsSug328, AsSug254, and DMA^V^, were not detected using HPLC- ESI-Q-TOF-MS. The arsenic compounds (% of Total As) detected using the anion-exchange column were as follows: AsSug328 and AsSug254 (96.3%), DMA^V^ (1.0%), and unidentified arsenic (0.6%) (Table 2).

**Table 2:** Detected peaks of arsenic species in artificial digestive system.

Sample	Percentage of Total As (%)					Recovery rate of HPLC (%)
	peak I (AsSug328 and AsSug254)	II (DMA^V^)	III (unidentified)	IV (thio-AsSug254)	V (thio-AsSug328)	
A	96.3	1.0	0.6	N.D.	N.D.	98
B	93.7	1.0	0.5	N.D.	N.D.	95
C	93.9	1.0	0.7	N.D.	N.D.	96
D	11.5	0.4	1.2	6.2	73.8	93
E	83.8	0.9	2.0	1.0	11.4	99

A: AsSug328 solution used in this study.

B: Sample A treated with artificial gastric juice for 4 h.

C: Sample B treated with artificial bile-pancreatic juice for 0.5 h.

D: Sample C treated with enteric bacteria solution for 24 h.

E: Sample D to which hydrogen peroxide solution was added.

The As compounds were separated by anion-exchange column chromatography using a PRP-X100 column.

Percentage of Total As (%) = (speciation As/Total As) × 100.

Recovery rate of HPLC (%) = (sum of speciation As/Total As) × 100.

N.D.: not detected.

In the HPLC-ICP-MS analysis using the cation-exchange column of NN614, the recovery rate of As [(sum of speciation As/Total As) × 100] was 97%. Three peaks were detected in sample A by using cation-exchange column. These peaks were identified as AsSug328, AsSug254, and DMA^V^ by HPLC-ESI-Q-TOF-MS. The arsenic compounds (% of Total As) detected using cation-exchange column were as follows: AsSug328 (80.8%), AsSug254 (14.7%), and DMA^V^ (1.5%).

The speciation values adopted for AsSug328 and AsSug254 were those obtained using the cation-exchange column and the values for DMA^V^ and unidentified arsenic were those obtained using the anion-exchange column. The AsSug328 reagent used in this study consisted of AsSug328 (80.8%), AsSug254 (14.7%), DMA^V^ (1.0%), and unidentified arsenic compounds (0.6%).

### 3.2 Change in the chemical form of AsSug328 after artificial gastrointestinal digestion

**Fig. 2:**
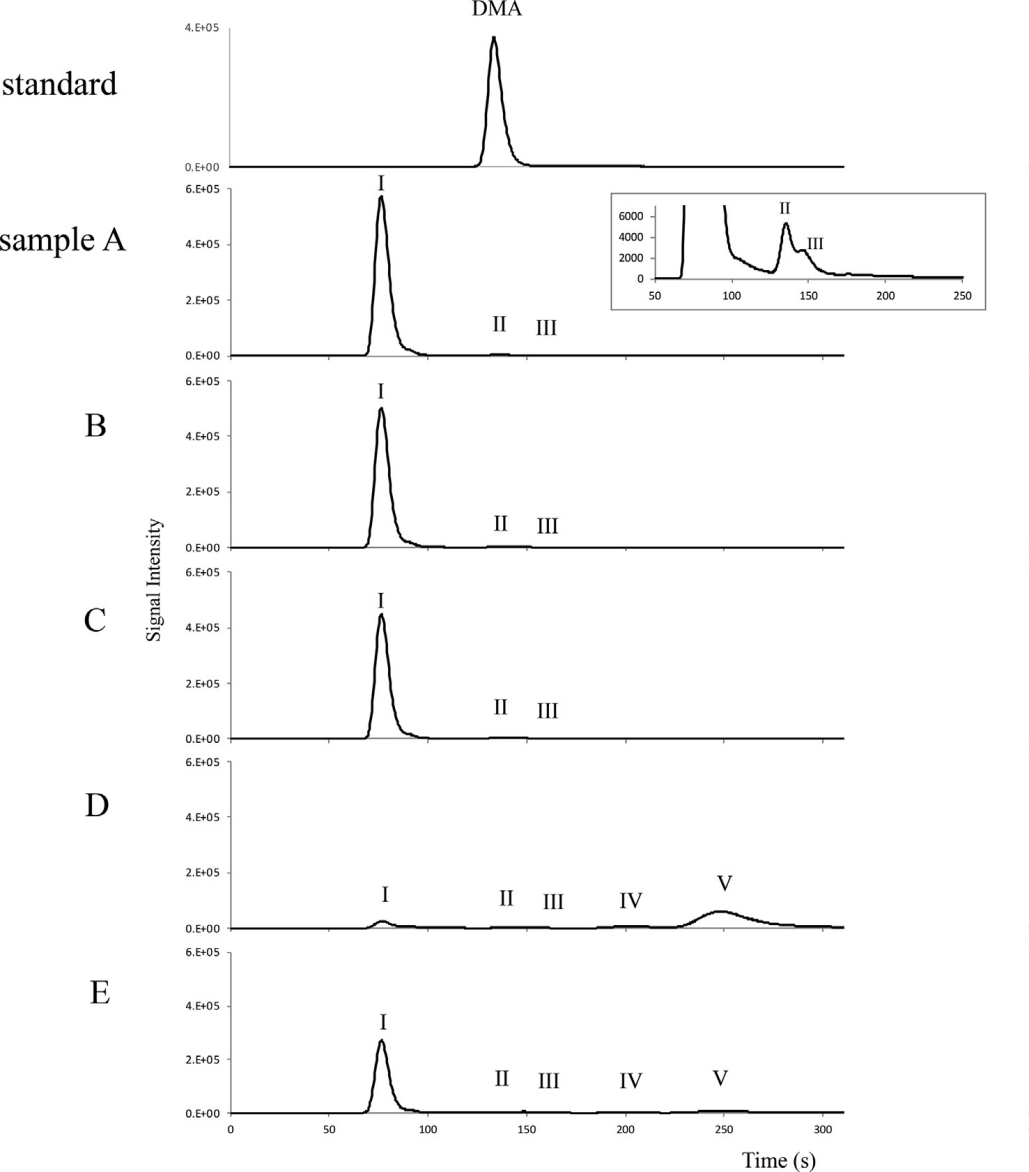
Chemical conversion of AsSug328 treated with artificial gastrointestinal juice, enteric bacteria solution, and hydrogen peroxide solution, as determined by HPLC-ICP-MS. The arsenic compounds were separated by anion-exchange column chromatography on a PRP-X100 column. A: AsSug328 solution used in this study. B: Sample A treated with gastric juice for 4 h. C: Sample B treated with bile-pancreatic juice for 0.5 h. D: Sample C treated with enteric bacteria solution for 24 h. E: Sample D with hydrogen peroxide solution added.

In all the samples from the digestive step, the recovery rate of As from the HPLC column was more than 90%, when the anion-exchange column, PRP-X100, was used (Table 2), and was more than 88%, when the cation-exchange column, NN-614, was used. Arsenic was not detected in the artificial digestive juice and enteric bacteria solution. After treatment with gastric juice (sample B) and bile-pancreatic juice (sample C), the pattern of HPLC-ICP-MS chromatograms did not change (Fig. 2). After the final incubation with enteric bacteria solution (sample D), the area of peak I (AsSug328 and AsSug254) was drastically decreased and the two peaks of the new arsenic compounds (peaks IV and V) were detected in the eluate from the anion-exchange PRP-X100 column (Fig. 2).

**Fig. 3:**
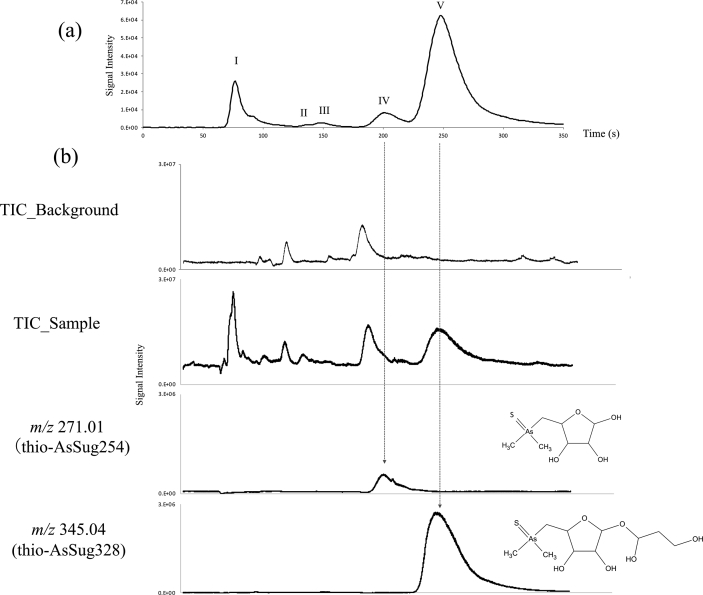
Chemical conversion of AsSug328 treated in tandem with gastric juice, bile-pancreatic juice, and intestinal bacteria (sample D). Chromatograms of sample D as determined by (a) HPLC-ICP-MS and (b) HPLC-ESI-Q-TOF-MS using anion-exchange column chromatography on a PRP-X100 column. The background is for sample D, without AsSug328.

The HPLC- ESI-Q-TOF-MS analysis revealed that peak IV consisted of an ion with an *m/z* of 271.01. This was determined to be C_7_H_16_AsO_4_S and was identified as thio-AsSug254 (Fig. 3). Peak V consisted of an ion with an *m/z* of 345.04. It was determined to be C_10_H_21_AsO_6_S and was identified as thio-AsSug328 (Fig. 3).

**Fig. 4:**
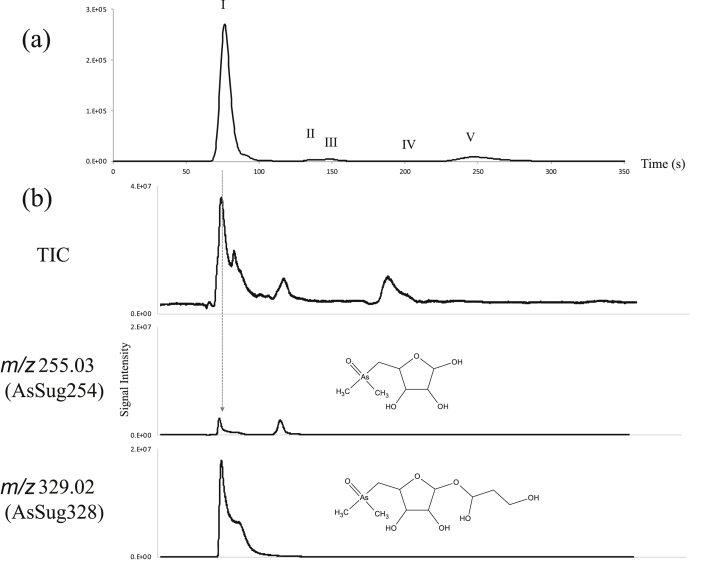
Chromatogram of sample E after H_2_O_2_ was added to the enteric bacteria-treated sample, as determined by (a) HPLC-ICP-MS and (b) HPLC-ESI-Q-TOF-MS using anion-exchange column chromatography on a PRP-X100 column.

In sample D, the elution time of DMA^V^ in the analysis using cation-exchange column, NN-614, almost overlapped with those of thio-AsSug328 and thio-AsSug254. When 0.1 mL of 30% (w/v) H_2_O_2_ was added to 0.9 mL of the enteric bacteria-treated samples (sample E), the peaks IV (thio-AsSug254) and V (thio-AsSug328) almost disappeared, and the peak I (AsSug328 and AsSug254) became more prominent than earlier (Fig. 4).

## 4 Discussion

Our results show that artificial gastrointestinal digestion converted AsSug328 and 254 into thio-AsSug328 and thio-AsSug254, respectively. However, no formation of DMA^**V**^ was detected. Peak III (unidentified As) was included in the AsSug328, used in this study. There is a possibility of the presence of a synthetic intermediate or a resolution product of AsSug328. Peak III increased as a result of the digestion process. However, the increase was negligible and we could not interpret from this increase that the artificial digestive system contributed to the disintegration.

The thio-arsenic compound was only produced after treatment with the enteric bacteria solution. Alava *et al.* [21] reported that 63% of As^III^ was transformed into As^V^ and 1.1% of monomethylmonothioarsenate (MMMTA^V^) after 48 h incubation with the *in vitro* colon suspension collected from the Simulator of the Human Intestinal Microbial Ecosystem (SHIME) reactor. The *in vitro* colon microbiota of SHIME was obtained from a 29-year-old male volunteer who had no history of antibiotic treatment in the past six months before the study [22]. The background of the enteric bacteria donor in our study was similar to that in the study by Alava *et al* [21]. The composition of intestinal flora depends not only on the history of antibiotic treatment but also on gender, age, and health condition [23, 24, 25, 26, 27]. Because the intestinal flora was obtained from healthy and young male adults in both these studies, their main constituents might be similar. In addition, Yoshida *et al.* [28] reported that urinary DMMTA^**V**^ was produced by intestinal bacteria following oral administration of DMA^**V**^ to rats. Therefore, thio-metabolites can be produced by intestinal bacteria of mammals.

Previous studies have shown that the main urinary metabolite in volunteers who ingested AsSug328 is DMA^**V**^, and that oxo- and thio-dimethylated arsenic compounds, for example, oxo-dimethylarsinoylethanol and DMMTA^V^, can only be detected in small amounts [11, 12]. The present study indicates that artificial gastrointestinal digestion of AsSug, which is the major component of seaweeds, does not produce dimethylated arsenic metabolites, especially DMA^**V**^. Therefore, AsSug will be absorbed in the intestinal tract after its sugar moiety is partially decomposed. They are then possibly metabolized to DMA^**V**^ in the liver and subsequently excreted through urine.

It has previously been shown that DMA^V^ promotes bladder cancer in rats [29]. In the experiment using rats, a small fraction (0.2%–0.3% of the intravenous dosage volume) of DMA^V^ in the bloodstream was excreted into the bile [30]. The DMMTA^V^ that was converted from DMA^V^ by enteric bacteria was absorbed into the bloodstream through the intestine and finally excreted into the urine [31]. Naranmandura *et al.* demonstrated that DMMTA^V^ is one of the most toxicologically potent arsenic species, relevant to arsenic-induced carcinogenicity in the urinary bladder [32]. These researches indicate the health risk of DMA^V^ arising from AsSug. It is essential to highlight the importance of decay of AsSug into DMA^V^ and the urinary excretion course of AsSug metabolites in evaluating the health risk of the organic arsenic ingested through marine products.

In conclusion, our study demonstrates that *in vitro* digestion of arsenic compounds present in seaweeds, using juices and enteric bacteria solution that mimic the *in vivo* digestion process, could provide vital information about the actual digestion of such compounds in the human gut. This would be helpful in assessing the toxicity and carcinogenicity of seafood.

## Declarations

### Author contribution statement

Akihisa Hata: Conceived and designed the experiments; Performed the experiments; Analyzed and interpreted the data; Wrote the paper.

Momoko Hasegawa, Yuki Otomo: Performed the experiments.

Takenori Yamauchi, Yuko Yamano Analyzed and interpreted the data.

Motofumi Miura: Contributed reagents, materials, analysis tools or data.

Kenzo Yamanaka: Contributed reagents, materials, analysis tools or data; Wrote the paper.

Noboru Fujitani, Ginji Endo: Conceived and designed the experiments.

### Funding statement

This work was supported in part by a Grant-in-Aid for Young Scientists (B) (26860442) from the Ministry of Education, Culture, Sports, Science and Technology of Japan.

### Competing interest statement

The authors declare no conflict of interest.

### Additional information

No additional information is available for this paper.
